# Polyethylene terephthalate (PET) primary degradation products affect c-di-GMP-, cAMP-signaling, and quorum sensing (QS) in *Vibrio gazogenes* DSM 21264

**DOI:** 10.1128/spectrum.00181-25

**Published:** 2025-06-09

**Authors:** Lena Preuss, Malik Alawi, Albert Dumnitch, Ly Trinh, Wolfgang Maison, Nils Burmeister, Anja Poehlein, Rolf Daniel, Christel Vollstedt, Wolfgang R. Streit

**Affiliations:** 1 Department of Microbiology and Biotechnology, University of Hamburg 14915, Hamburg, Germany; 2 Bioinformatics Core, UKE Hamburg, Hamburg, Germany; 3 Department of Chemistry, University of Hamburg 14915, Hamburg, Germany; 4 Department of Genomic and Applied Microbiology, Georg-August University of Göttingen, Göttingen, Germany; Molecular and Computational Biosciences and Biotechnology, Migal-Galilee Research Institute, Kiryat Shmona, Israel

**Keywords:** *Vibrio *sp. biofilms on plastics, plastisphere, polyethylene terephthalate (PET), bis(2-hydroxyethyl) terephthalate, mono(2-hydroxyethyl) terephthalate (MHET), phages, PETase

## Abstract

Global plastic pollution in oceans and estuaries is increasing rapidly, and it is well known that bacteria colonize plastic particles of all sizes. *Vibri*o spp. are frequently found as part of the plastisphere. We recently showed that *Vibrio gazogenes* DSM 21264 harbors a promiscuous esterase designated PET6. We now provide evidence that the *pet6* gene is expressed under a wide range of environmental conditions in its native host. However, in PET- and PE-grown biofilms, the *pet6* gene expression was not affected by the type of surface. The *pet6* transcription was sufficient to allow enzyme production and release of µM amounts of mono-(2-hydroxyethyl) terephthalate (MHET) and terephthalic acid (TPA) already after 24 hours of incubation on the PET foil. Notably, the highest *pet6* gene transcription was observed in planktonic lifestyle in the presence of bis(2-hydroxyethyl) terephthalate (BHET), one of the primary degradation products of PET. BHET was further hydrolyzed by PET6 and UlaG, a lactonase that had not been known to be involved in BHET degradation. Elevated concentrations of BHET affected the major signaling circuits involved in bacterial quorum sensing (QS), c-di-GMP, and cAMP-CRP signaling. This resulted in failure to form biofilms, synthesis of the red pigment prodigiosin, and altered colony morphologies. While BHET had a very wide impact, TPA interfered mainly with the bacterial QS by attenuating the expression of the CAI-I autoinducer synthase gene. These observations imply a potential role of BHET and TPA as nutritional signals in *Vibrio gazogenes* and that may affect its growth and survival in the plastisphere.

**IMPORTANCE:**

This study provides the first evidence that *Vibrio gazogenes* DSM 21264 secretes an active polyethylene terephthalate (PET) hydrolase and degrades the polymer using PET6 when growing in biofilms on foils and microplastic particles. The study further provides evidence that the primary PET degradation products bis(2-hydroxyethyl) terephthalate (BHET) and terephthalic acid (TPA) may have a profound impact on the global QS, c-di-GMP, and cAMP-CRP signaling of *V. gazogenes* and its capability to colonize plastic particles in the marine environment.

## INTRODUCTION

Global plastic pollution has reached an alarming and unprecedented level in the recent decade. Recently, it was estimated that a minimum of 8–10 million tons of plastic is entering the oceans annually (1–5). While most of the plastic enters the environment as larger floating fragments, weathering results in the production of smaller particles (micro-, nano-, and pico-plastics) with sizes less than 5 mm in diameter (6, 7). These particles and the additives contained in them are believed to have a negative impact on all ecological niches and their biodiversity (8, 9). Ultimately, they will also affect human health and wellbeing (10).

The majority of the fossil fuel-based and synthetic polymers which end up in the environment are polyethylene (PE), polypropylene (PP), polyvinylchloride (PVC), polyethylene terephthalate (PET), polyurethane (PUR), polystyrene (PS), and polyamide (PA) only, being produced at lower quantities (11). While for most of these polymers no enzymes and microorganisms are known to degrade them, the degradation of PET is well understood (https://www.pazy.eu) (12). PET is degraded by promiscuous and secreted esterases, lipases, or cutinases that all have broad substrate specificity and are members of the E.C. classes 3.1.1.- (11, 13, 14). In addition to these studies, recently, much effort has been made to analyze and characterize the phylogenetic makeup of microbial communities associated with plastic particles found in the marine environment. The microorganisms colonizing plastic particles (i.e., the plastic microbiota) have been termed “plastisphere” (15–18). These studies indicate that growth on and the initial colonization of plastic surfaces depends on the chemical and the physical properties of the various polymers. Thereby, the surface charge, roughness, and the different chemical compositions of additives play key roles during the attachment and growth of the plastic colonizing microbiota (16, 19).

Notably, simple colonization does not imply any biodegradation (6). The microbial communities observed are highly diverse in their phylogeny, and bacteria affiliated with the genus *Vibrio* are often observed on plastic particles (18, 20–24). *Vibrio* species are ubiquitously occurring gram-negative bacteria that are mainly found in marine environments where they are considered to be key players in carbon and nitrogen cycling (25, 26). There are more than 100 known *Vibrio* species, of which few are either human or fish pathogens (e.g., *Vibrio cholerae*, *Vibrio parahaemolyticus, Vibrio alginolyticus, and Vibrio vulnificus* (27–29).

Recently, we have shown that the marine organism *V. gazogenes* DSM 21264 (from here on DSM 21264) harbors a gene encoding a promiscuous esterase designated PET6 in its 4.6 Mbp genome (30, 31). DSM 21264 (synonym PB1, ATCC 29988) is a gram-negative and nonpathogenic bacterium. It was isolated from sulfide-containing mud collected from a saltwater marsh and produces prodigiosin, a red pigment (32). The species is globally occurring and typically found in estuaries. The *pet6* gene is encoded by ORF AAC977_05355. The heterologously expressed protein hydrolyzes amorphous PET and other substrates including short-chain fatty acid esters (30, 31), and its activity is salt-dependent. PET hydrolysis catalyzed by recombinant PET6 is, however, rather low compared to other known enzymes used in industrial processes like the well-characterized IsPETase or LCC (31). While much information was gathered on the enzyme structure and biochemical function of PETases, only a few studies focused on the expression of the enzyme within the native host. *Ideonella sakaiensis* is among the only organisms for which the PET metabolism is well understood (33). With respect to DSM 21264, it was not known under which conditions the *pet6* gene is transcribed in its native host and if the native enzyme would result in PET degradation in the environment. Within this manuscript, we provide the first evidence that the *pet6* gene is transcribed at low levels under various environmental conditions. The *pet6* gene expression is, however, not affected by the presence of PET, PE foil, or PET powder.

Instead, BHET, a primary PET degradation product, affects *pet6* transcription at mM concentrations. Further, our data imply that BHET is a nutritional signal affecting the c-di-GMP, the cAMP-CRP, and QS-dependent signaling pathways in DSM 21264. These three signaling pathways are essential to lifestyle transitions from motility, attaching to a surface, and forming biofilms in the plastisphere.

## RESULTS

### DSM 21264 forms patchy biofilms on PE and PET independent from the surface and releases micromolar concentrations of MHET and TPA

**Fig 1:**
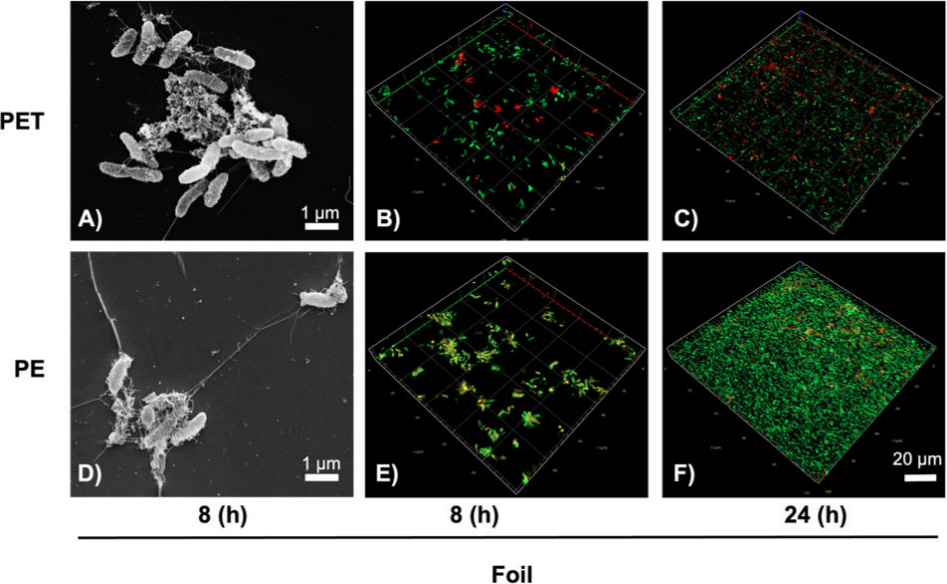
Scanning electron microscopy (SEM) (**A, D**) and confocal laser scanning microscopy (CLSM) images (**B, C, E, F**) of *V. gazogenes* DSM 21246 biofilms grown on PET and PE foil. SEM images and CLSM images (A), (**B**), and (D); (**E**) show first attachment of cells after 8 hours of incubation in the ASW medium at 22°C of incubation on PET foil (A) and (B) and PE foil (D) and (E). CLSM images (C) and (F) show biofilm formation of DSM 21264 on PET foil (C) and PE foil (F) after 24 hours of incubation. Cells were stained using LIVE/DEAD stain.

Since we had earlier shown that DSM 21264 produces a PET-active hydrolase, designated PET6 (30, 31), we wanted to know if the organism forms biofilms on PET foil and actively degrades PET. Further, we asked if the *pet6* gene is expressed at significant levels. Therefore, we inoculated DSM 21264 in artificial seawater medium (ASWM) with PET or PE foil for biofilm formation and potential degradation. Since no PE-degradative genes have been reported in gram-negative bacteria, PE foil was used as a control. In these biofilm experiments, cells attached in general at very low frequencies, and only thin single-cell layer biofilms were formed after 10 and 180 days (Fig. 1; Fig. S1). Therefore, we used ASWM supplemented with tryptone and yeast extract 1% (wt/vol) in further tests to promote growth and biofilm formation. Under these conditions, DSM 21264 attached within few hours and formed patchy biofilms on both plastic surfaces, whereby the biofilms formed on PE were less patchy than those on PET (Fig. 1). Biofilms formed on PE and PET had an average thickness of 2–3 μm, equaling one cell layer (Fig. 1).

**Fig 2:**
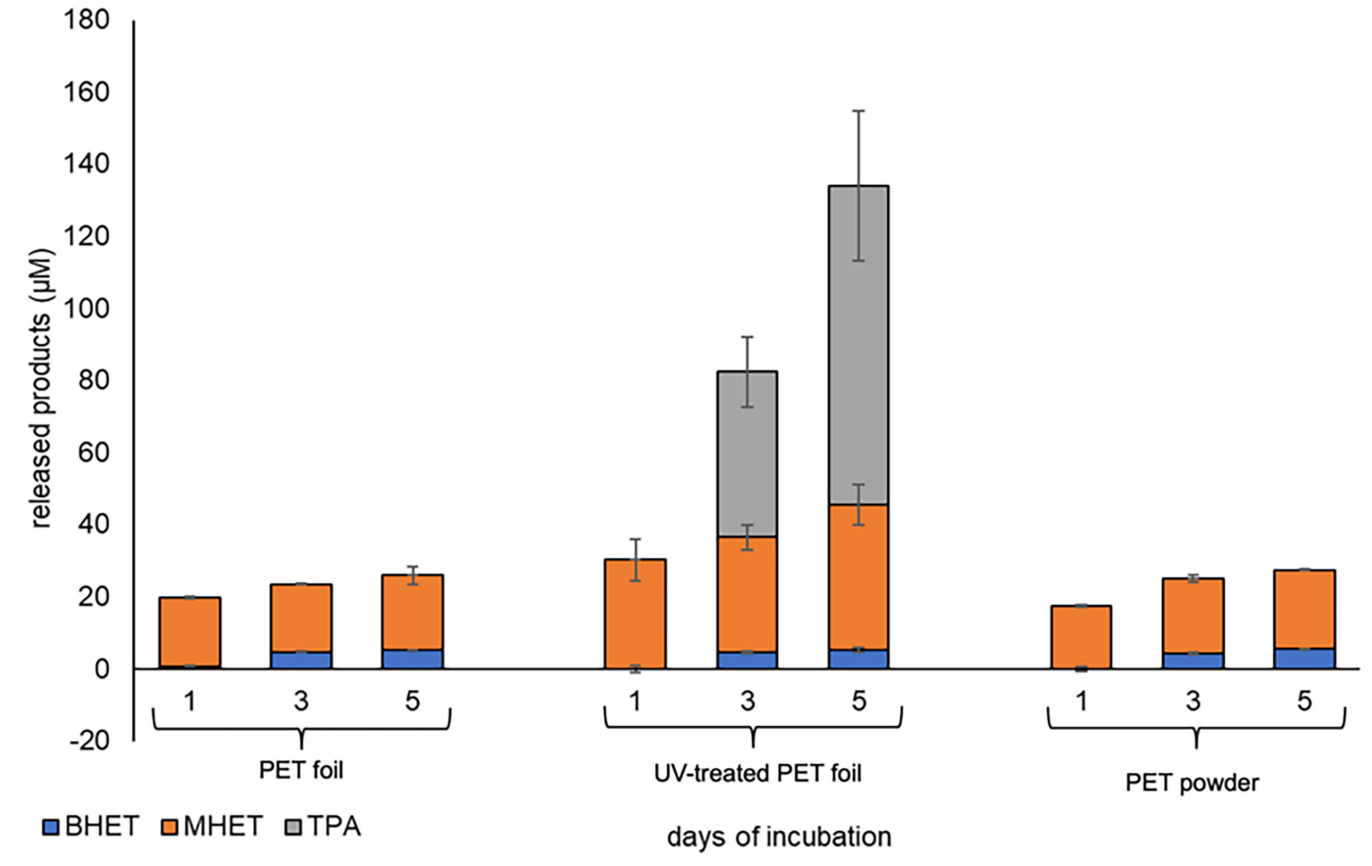
PET degradation products observed in supernatants of *V. gazogenes* DSM 21264 biofilms grown on PET foil (non-treated), UV-treated foil, and PET powder. *V. gazogenes* DSM 21264 was grown in the ASW medium with reduced carbon source. Supernatants were collected after 1, 3, and 5 days, and the degradation products were measured using UHPLC and as described in the Material and Methods section. Data were normalized and corrected to *E. coli* DH5α growing on PET foil and powder. Data are expressed as mean values of three independent replications, and bars represent the standard deviations.

SEM images showed that the cells on PET and PE were interconnected by fiber- and net-like structures with a length of 4–10 μm. However, the net-like structures were more prominent on PE surfaces (Fig. 1). Additional tests with air plasma-treated PET and PE foil were conducted (34). Air plasma treatment leads to the formation of oxygen functionalities and thus an increase in surface polarity. It typically gives a potential degradative enzyme better access to the polymer fibers (35). However, in these tests, only minor differences were observed with respect to biofilm formation (Fig. 1; Fig. S1). Notably, when we grew DSM 21264 on PET foil or powder, we detected micromolar amounts of BHET, MHET, and TPA in the biofilm and culture supernatants already after 24 hours (Fig. 2).

### Transcriptome analysis identifies keys to PET6 gene expression

**Fig 3:**
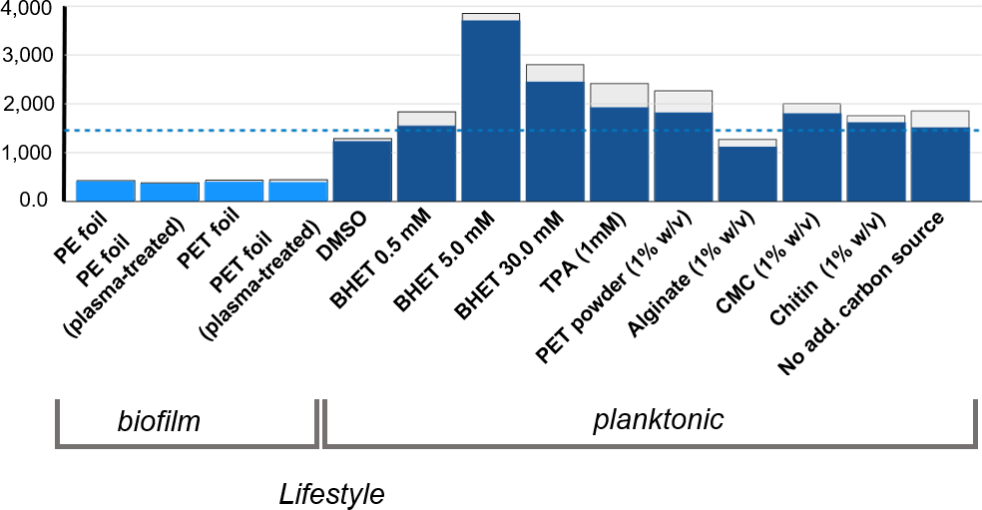
Transcription level of the *pet6* gene in relation to all 4,117 genes transcribed in DSM 21264. Blue bars indicate the ranking of *pet6* depending on all genes transcribed. Each data set represents the mean data from three independent biological replicates. Standard deviations are indicated as light gray bars on top of the colored bars. The blue dotted line indicates the mean transcription level of all 4,117 genes in all 42 experiments.

Based on the above-made observations, we further asked if the *pet6* gene (AAC977_05355) was expressed and if surface attachment to PET or PE resulted in major transcriptional changes. To address these questions, we mainly used transcriptome studies with the goal of obtaining insight into the overall gene expression pattern of DSM 21264 in response to PET or other substrates during life in biofilms and planktonic cultures. In total, we analyzed 14 different environmental parameters and/or carbon sources. Each experiment was repeated three times, resulting in 42 RNAseq data sets that have been submitted to the European Nucleotide Archive (ENA). They are publicly available under accession PRJEB80907. Obtained reads were mapped against the genome of DSM 21264. For this purpose, we established a high-quality genome sequence of DSM 21264 that encompasses two chromosomes coding for 4,159 genes. Chromosome 1 (CP151640) codes for 3,121 genes and chromosome 2 (CP151641) for 1,038 genes. The newly established genome sequence is available under accession numbers CP151640 and CP151641 at NCBI/GenBank. An overview of the data sets reads obtained, the percentage of reads mapped to the genome, and other parameters together with the level of *pet6* gene expression is given in Table S2; Fig. 3.

### PET6 is weakly expressed in biofilms grown on PET and PE foils

When we compared PET-grown biofilms versus PE-grown biofilms, the expression *of pet6* was low and ranked around 25% of the weakest transcribed genes. Fig. 3 summarizes the level of expression of *pet6* under the biofilm conditions tested and in relation to the genome-wide expression of all genes. Plasma treatment of the PET or PE foil had no significant impact on the low level of *pet6* gene expression (Table S1; Fig. 3). This observation is in line with the slow degradation of the polymer in biofilm cultures, as described above (Fig. 2). In addition, we noticed that in all biofilm experiments, the transcription of *ompU* was among the highest transcribed genes. OmpU is an outer membrane protein that has for *V. cholerae* been described to be important for biofilm formation (36), implying that OmpU possibly is involved in surface colonization. Furthermore, only four genes were differentially expressed in all biofilm studies (Table S1 and S3). Highest log2-fold changes were detected for a glutathione peroxidase and an alkyl hydroxide peroxidase, both involved in hydrogen peroxide scavenging (37). In addition, the *adhE* gene, which is involved in ethylene glycol (EG) metabolism, was significantly upregulated in PET biofilms but not in PE-grown biofilms (Table S1). To further verify the increased expression of *adhE,* a reporter fusion was constructed fusing the promoter with the amcyan gene in pBBR1-MCS-1 and mobilized into DSM 21264 (Table 1). Using the *P_adhE::amcyan* transcriptional reporter gene fusion in DSM 21264, we were able to verify that *adhE* was twofold upregulated in the presence of 5 mM EG (Fig. S5).

**TABLE 1:** Bacterial strains and plasmids used in this study

Strain or plasmid	Relevant trait(s)	Source or reference
Strains		
*E. coli* DH5α	F^-^ ɸ80d*lacZ*ΔM15 Δ(*argF-lacZYA*) U169 *endA1 hsdR17* (r_K_^-^. m_K_^-^) *supE44 thi-1 recA1 gyrA96 relA1*	(38)
*E. coli* WM3064	*thrB1004 pro thi rpsL hsdS lacZ*ΔM15 RP4–1360 Δ (*araBAD*)*567* Δ*dapA1341*::[*erm pir*(wt)]	Metcalf, University of Illinois, Urbana-Champaign, USA
*E. coli SHuffle T7*	*huA2 lacZ::T7 gene1 [lon] ompT ahpC gal* *λatt::pNEB3-r1-cDsbC (SpecR) lacIq)* *ΔtrxB sulA11 R(mcr-73::* *miniTn10—TetS)2 [dcm] R(zgb-210* *::Tn10—TetS) endA1 Δgor ∆(mcrC-mrr)114::IS10*	NEB, Frankfurt am Main, Germany
*E. coli* BL21 (DE3)	*F−. ompT. hsdS B (rB− m B−) gal. dcm. λDE3*	Novagen/Merck, Darmstadt, Germany
		
*V. gazogenes* DSM 21264	Wild-type strain	DSMZ, Braunschweig, Germany
		
Plasmids		
pBBR1MCS-1	Broad host range vector. Low copy no.; Cm^r^	(39)
pBBR1-MCS-1::*amcyan*	*pBBR1MCS-5* carrying the *amcyan gene* in the MCS between *Xba*I and *Bam*HI restriction site	This work
pBBR1-MCS-1::a*dh*E*::amcyan*	*pet6*::*amcyan* reporter fusion in *pBBR1MCS-5* between *SacI and XbaI* restriction site in front of *amcyan*	This work
pET21a(+)	Expression vector. *lacI*. Amp^R^ T7-*lac*-promoter. C-terminal His-tag coding sequence	Novagen/Merck (Darmstadt, Germany)
pET21a(+)::*ulaG*	1,056 bp insert in pET21a(+) coding for UlaG	This work
pET28a(+)	Expression vector. *lacI*. Kana^R^ T7-*lac*-promoter. C-terminal His-tag coding sequence	Novagen/Merck (Darmstadt, Germany)
pET28a(+)::*pet6*	894 bp insert in pET28a(+) coding for PET6	(31)

**Fig 4:**
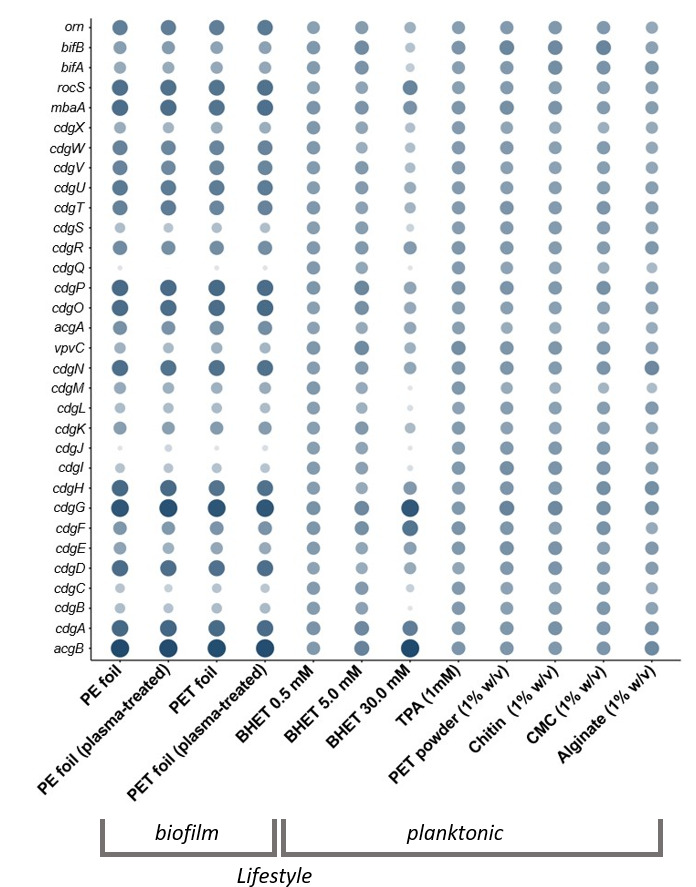
Relative transcription level of c-di-GMP-signaling-related genes in DSM 21264. The circle size and color correlate with the normalized transcription level. The color intensity and size of the circles are adjusted to logarithmized values (log_value) ranging from −2 to 4 and according to the mapped reads. CdgA-CdgN are diguanylate cyclases (DGC), CdgO-CdgX are phosphodiesterases (PDE). *bifA* and *bifB* code for bifunctional genes, and Orn is an oligoribonuclease. Each data point is a mean value of three independent experiments for each of the 12 conditions shown.

**Fig 5:**
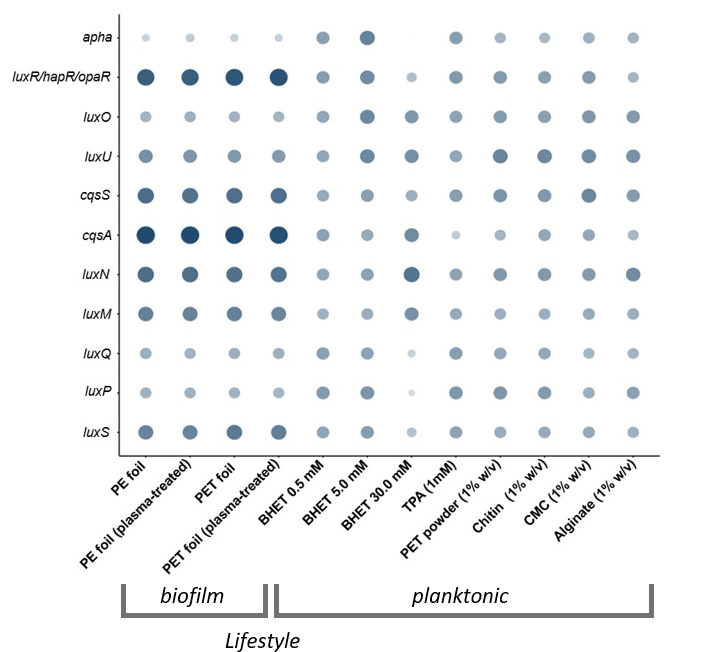
Relative transcription level of essential quorum sensing (QS)-related and differentially regulated genes in DSM 21264. The circle size and color correlate with the normalized transcription level. The color intensity and size of the circles are adjusted to logarithmized values (log_value) ranging from −2 to 4 and according to the mapped reads. *luxS*, *luxM,* and *cqsA* code for autoinducer synthases, LuxP is annotated as a periplasmatic binding protein, and LuxQ, LuxN, and CqsS are autoinducer sensor kinases. AphA is the low-cell density regulator, and HapR is the high-cell density regulator. Each data point is a mean value of three independent experiments for each of the 12 conditions shown.

**Fig 6:**
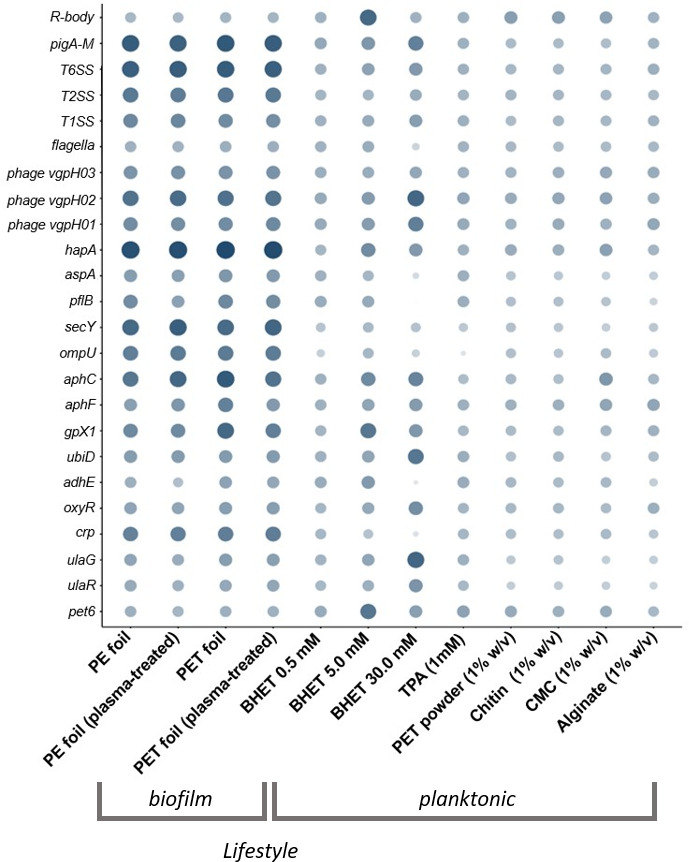
Relative transcription level and changes of differentially regulated major genes and gene clusters in DSM 21264. The circle size and color correlate with the normalized transcription level. The color intensity and size of the circles are adjusted to logarithmized values (log_value) ranging from −2 to 4 and according to the mapped reads. R bodies belong to the Rebb family gene cluster, and the pig gene cluster is responsible for the synthesis of prodigiosin. hapA is M4 metallopeptidase annotated as vibriolysin, *aspA* encodes an aspartate ammonia-lyase, and *pflB* codes for a formate C-acetyltransferase. Each data point is a mean value of three independent experiments for each of the 12 conditions shown.

Since PET and PE are no natural substrates for bacteria, we further asked if other natural polymers would stimulate *pet6* gene expression. Therefore, we added alginate, chitin, and carboxymethyl cellulose (CMC) (1% wt/vol) to planktonic cultures and assayed the transcriptomes after 24 hours of aerobic growth. As expected in the planktonic cultures, a largely different pattern of gene transcription was observed compared to the biofilm conditions (Table S1; Fig. 4 to 6; Fig. S2). Since under all tested conditions a number of reads were mapped to the *pet6* gene, it appears to be constitutively expressed (Fig. 3). In general, the transcription level of *pet6* was low and close to 0.4% of the transcription observed for *rpoD* (Table S2). In summary, the data imply that *pet6* gene expression is not strongly activated by any of the added natural polymers or by PET powder (1% wt/vol) containing cultures. However, the expression was significantly higher in the planktonic cultures compared to the biofilm cultures (Fig. 3).

### *Pet6* and *ulaG* transcription are induced by BHET in DSM 21264

Since PET6 hydrolyzes PET, we further asked if DSM 21264 would be able to metabolize BHET and use it as a sole carbon and energy source, and if it would affect *pet6* gene expression. To address these questions, additional experiments in liquid media were performed challenging DSM 21264 with BHET at concentrations of 0.5, 5.0, and 30 mM. The growth of the strain was not affected by lower BHET concentrations (0.5 mM and 5.0 mM) after 24 h of incubation (Fig. S8). Since higher concentrations of BHET precipitate, measurement of OD_600_ was not valid. Testing 30 mM of BHET on agar plates determined less colony formation than other tested concentrations, implying reduced growth (Fig. S6). The overall gene expression profile using RNAseq was tested for the respective BHET concentrations. As controls, we incubated DSM 21264 in the presence of 1.0 mM TPA, DMSO (0.5% vol/vol), and PET powder (1% wt/vol) in liquid cultures. Table S2 summarizes the data obtained for all RNAseq experiments. The volcano plots highlight major changes in gene expression (Fig. S2). A detailed analysis identified a large number of genes and operons differentially regulated (>log2-fold change of 2) in the presence of the different BHET concentrations, clearly indicating that DSM 21264 senses and adapts its metabolism to the presence of BHET (Table S1; Fig. 4 to 6). High concentrations (5.0 and 30 mM) of BHET had the most pronounced effects on the overall gene expression levels in DSM 21264 with >230 upregulated and >100 downregulated genes in the presence of 5.0 mM and >830 upregulated and >550 downregulated genes in the presence of 30 mM BHET compared to the DMSO or the TPA controls (Fig. S7). Our data imply that BHET is most likely initially metabolized by PET6, releasing MHET as the primary degradation product of BHET. This is in line with an almost fivefold (log2-fold change) increased transcription level of *pet6* (AAC977_05355) at 5 mM BHET and 1.8-fold increase at 30 mM. Surprisingly, the RNAseq data indicated that UlaG, a predicted metallo-ß-lactamase, is also involved in BHET metabolism. In the presence of 30 mM BHET, *ulaG* was most strongly transcribed with a 7.9-fold upregulation compared to the control. To further verify this novel role of UlaG, we expressed it in *E. coli* T7 SHuffle and used the recombinant protein to hydrolyze BHET. In these tests, the promiscuous enzyme was able to cleave BHET at slow but significant rates (Fig. S3). Further UHPLC measurement confirmed that MHET is the main and initial degradation product of DSM 21,264 cells grown on BHET (Fig. S4). While MHET is converted to EG and TPA, DSM 21264 was neither able to grow on TPA and EG as the sole carbon and energy source nor on BHET. It does not encode any mono- and dioxygenases involved in aromatic ring cleavage.

### BHET and TPA affect the central circuits involved in c-di-GMP, cAMP-CRP, and QS signaling in DSM 21264

During the above-described growth experiments using BHET as a substrate, we noticed major phenotypic changes in DSM 21264 affecting colony morphology, biofilm formation, prodigiosin biosynthesis, and others (Fig. S6).

BHET altered colony morphology already at rather low concentrations (0.5 mM). Colonies were less structured and had smooth edges. Higher concentrations of BHET resulted in the disappearance of the red pigment prodigiosin. Biofilm formation was disturbed at 0.5 mM, and no solid biofilms were formed at 10 mM BHET present in the medium (Fig. S6).

These phenotypic observations implied a larger impact of BHET on the metabolism and main regulatory circuits of DSM 21264 in planktonic cultures. To further elucidate these effects, we analyzed our transcriptomic data with respect to the influence of BHET on regulatory pathways linked to QS, cAMP-CRP signaling, and c-di-GMP signaling. These pathways were chosen as they are known to have a wider impact on biofilm formation, motility, secretion, secondary metabolite production, and others.

Most notably, 30 mM BHET strongly affected the transcription of genes essential for c-di-GMP biosynthesis. The signal c-di-GMP is synthesized by diguanylate cyclases degraded by phosphodiesterases. DSM 21264 harbors at least 16 possible c-di-GMP cyclases/phosphodiesterases in its genome. The majority of the diguanylate cyclases were strongly downregulated in their transcription in the presence of high concentrations of BHET, while they were strongly upregulated in the presence of low concentrations of BHET or other carbon sources (Fig. 4). The most strongly attenuated genes coding for diguanylate cyclases were *cdgB, C, I, J,* and *M* (log2-fold change < –4.5). On the contrary, *cdgG* and *acgB* were strongly upregulated in the presence of increased BHET concentrations (log2-fold change 3.5 and 5) (Fig. 4).

Further, we observed that BHET affected quorum sensing (QS) processes. In this context, transcription of the master QS regulator *aphA* was almost not detectable at 30 mM BHET, while it was well expressed at 0.5 and 5.0 mM BHET or in the presence of TPA in planktonic cultures (Fig. 5). HapR was also only weakly transcribed in the presence of 30 mM BHET. While it is well known that AphA and HapR are usually transcribed at opposite levels, no differences in transcription levels were visible neither in the presence of lower concentrations of BHET nor in the presence of TPA. These differences in expression were, however, clearly visible in all the biofilm experiments and in the planktonic cultures supplemented with natural polymers (Fig. 5).

Furthermore, TPA negatively affected the transcription of the autoinducer synthase gene *cqsA,* which is involved in the cholera autoinducer I biosynthesis (CAI-I) (Table S1; Fig. 5). Further, our data showed that the DSM 21264 autoinducer (AI) -I synthase, LuxM*,* and the cognate sensor LuxN, as well as the genes coding for the AI-2 synthase, *luxS*, the periplasmatic binding protein, LuxP, and the sensor kinase LuxQ were significantly affected in their transcription levels at 30 mM BHET (Fig. 5).

Finally, our data implied that BHET interfered with cAMP-CRP signaling. The catabolite activator protein transcription was almost completely turned off in the presence of 30 mM BHET (Fig. 6).

Besides the direct impact of 30 mM BHET on the above-mentioned regulatory circuits, 30 mM BHET strongly attenuated the transcription of motility genes, *hap*A, *aspE,* and *ompU*. On the contrary, the expressions of *ulaG, ulaR*, *aphC*, *aphF,* and the prodigiosin operon were strongly upregulated (Fig. 6).

Interestingly, among the highest upregulated genes in the samples supplemented with 30 mM BHET, several phage-related genes were observed. In total, DSM 21264 codes for three prophages (Phage 1, designated VGPH01, AAC977_19445 – AAC977_19700; Phage 2, designated VGPH02, AAC977_20360 – AAC977_20490; Phage 3, designated VGPH03, AAC977_09080 – AAC977_09210), which can be grouped into the class of Caudoviricetes. VGPH01 and VGPH02 are encoded on the smaller chromosome CP151641, whereas VGPH03 is part of the larger chromosome CP151640. All three phages were transcribed under biofilm conditions. In contrast, their transcription was generally low in planktonic cultures. However, in planktonic cultures, 30 mM BHET resulted in strong transcription of the gene clusters coding for VGPH01 and VGPH02 (Table S4; Fig. 6).

In summary, our data imply that BHET and TPA may have a wider effect on the metabolism of DSM 21264 and interfere with the interconnected pathways involved in c-di-GMP, QS, and cAMP-CRP signaling.

## DISCUSSION

Today, 125 PET-active enzymes are known, and most of these enzymes have been characterized very well with respect to their structures, functions, and catalytic activities on the synthetic polymer. These enzymes are generally secreted and promiscuous hydrolases belonging to the E.C. 3.1.-. (6, 11–14). However, only very few studies have analyzed the growth of bacteria on the PET foil using global RNAseq approaches (40–42). Despite these first studies, it is not clear if bacteria differentiate between the different types of synthetic polymers either as a surface to attach to or as a potential substrate for breakdown.

Our data suggest that only very few genes are differentially regulated when DSM 21264 is grown on PET versus the non-biodegradable PE, implying that most likely DSM 21264 is not able to differentiate between these two polymers. Also, the treatment with plasma did not affect this response. Interestingly, our data identified *ompU* as one of the most strongly transcribed genes when grown in biofilms (Table 2). OmpU is an outer membrane protein known to be an important adherence factor in *Vibrio cholerae* and an essential biofilm matrix assembly protein (36). Based on these earlier findings, it is likely that the DSM 21264 OmpU plays a major role in biofilm formation on plastic surfaces and life in the plastisphere.

**TABLE 2:** The 25 genes with the highest normalized counts in DSM 21264 grown in biofilms on PET and PE

8 hour biofilms				
#	PET foil		PE foil	
	Predicted function	Locus tag	Predicted function	Locus tag
1	*ompU*, porin	AAC977_02740	*ompU*, porin	AAC977_02740
2	*secY*, translocase	AAC977_01575	*secY*, translocase	AAC977_01575
3	*pflB,* acetyltransferase	AAC977_06195	*pflB*, acetyltransferase	AAC977_06195
4	r*poA*, RNA polymerase	AAC977_01600	*rpoA,* RNA polymerase	AAC977_01600
5	Dehydrogenase	AAC977_05600	Elongation factor	AAC977_11845
6	*fusA,* elongation factor	AAC977_11845	*rpoB,* RNA polymerase	AAC977_14125
7	*rpoB*, RNA polymerase	AAC977_14125	*rpoC*. RNA polymerase	AAC977_14120
8	Peptide synthetase	AAC977_13870	Peptide synthetase	AAC977_13870
9	*rpoC*. RNA polymerase	AAC977_14120	Cold-shock protein	AAC977_16695
10	*aspA,* ammonia-lyase	AAC977_00985	Dehydrogenase	AAC977_05600
11	^*a*^Metallopeptidase	AAC977_18400	*aspA*, ammonia-lyase	AAC977_00985
12	Cold-shock protein	AAC977_16695	Methyltransferase	AAC977_01345
13	Methyltransferase	AAC977_01345	Dehydrogenase	AAC977_02520
14	*tuf,* elongation factor	AAC977_13935	Elongation factor	AAC977_13935
15	ATP synthase	AAC977_15540	Acyl carrier protein	AAC977_10205
16	*tuf,* elongation factor	AAC977_14160	ATP synthase	AAC977_15540
17	l*uxR*, regulator	AAC977_02535	Elongation factor	AAC977_14160
18	Chaperone	AAC977_04160	^*a*^Metallopeptidase	AAC977_18400
19	Fumarate reductase	AAC977_01180	Chaperone	AAC977_04160
20	Chaperonin	AAC977_01145	Quinone reductase	AAC977_11710
21	Quinone reductase	AAC977_11710	Elongation factor	AAC977_13940
22	ATP synthase	AAC977_15530	ATP synthase	AAC977_15530
23	Acyl carrier protein	AAC977_10205	Dehydrogenase	AAC977_02525
24	Aldolase	AAC977_13010	ACP synthase	AAC977_10200
25	Pyruvate dehydrogenase	AAC977_02520	Nucleotidyltransferase	AAC977_02820

^*a*^ vibriolysin.

Only a few *Vibrio* species harbor PET6 homologs, but all of them share a very conserved core genome and have common regulatory networks (31, 43, 44). Within this setting, our data imply that *pet6* is expressed at low levels under most environmental conditions (Fig. 3), and increasing BHET concentrations affected the transcription of the gene.

**Fig 7:**
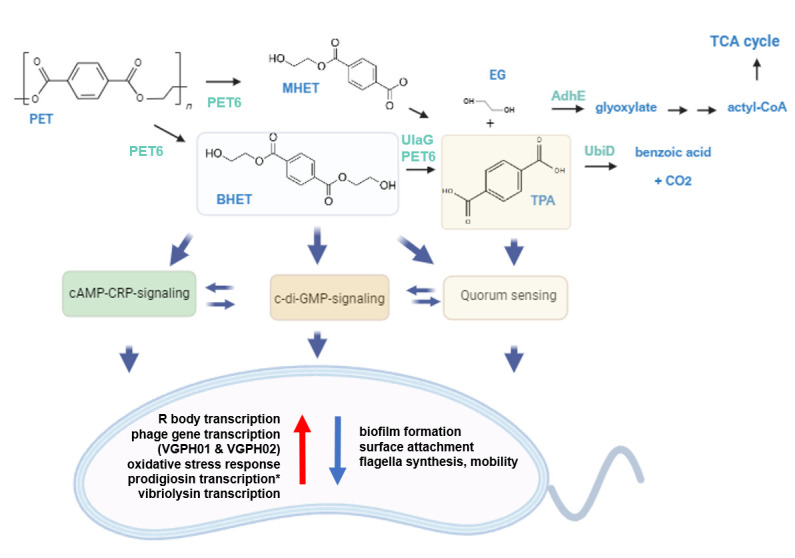
Possible PET degradation pathway in DSM 21264 and regulatory model affecting QS, cAMP-CRP, and c-di-GMP signaling through BHET and TPA. Proteins involved in enzymatic PET breakdown are in cyan color. PET and degradation products released are labeled in blue. Blue arrows indicate regulatory pathways affected by BHET and/or TPA in DSM 21264. Red and blue arrows inside the cell model indicate up- and downregulated pathways. *Transcription of the prodigiosin operon is strongly upregulated (log2-foldchange > 3) in the presence of high BHET concentrations, but synthesis of the pigment is inhibited.

Besides these observations, our study has further revealed that UlaG is involved in BHET metabolism. Previous work has already demonstrated that UlaG is a promiscuous metallo-beta-lactamase with a wide range of functions in bacteria and archaea (45, 46). We observed a strongly increased transcription of *ulaG* in the presence of high BHET concentrations. Additional BHET degradation assays confirmed this novel functional role of UlaG (Fig. S3; Fig. 7). *Ideonella sakaiensis* is the only organism for which the whole degradation pathway of PET is mostly understood. PET is degraded into MHET and EG and further transformed by the IsMHETase into TPA and EG. EG is then oxidized by an alcohol dehydrogenase to glycolaldehyde (GAD) and further metabolized by the aldehyde dehydrogenase to glycolic acid (GA) (47). Similar to the EG metabolism of *I. sakaiensis*, our data support the notion that *adhE* in DSM 21264 is involved in the metabolism of EG. This observation was supported by both RNAseq and experimental data (Fig. S5). Since *adhE* is transcribed at higher levels in PET biofilms, it is likely that this increase is caused already by the degradation of the polymer and the parallel BHET release (Fig. 7).

Additionally, the data from our study suggest that BHET and TPA are nutritional signals affecting QS, c-di-GMP, and cAMP signaling at micromolar concentrations (Table S1; Fig. 4 to 6). This hypothesis is based on 42 transcriptomic data sets of DSM 21264 that have given us insights into the response of this bacterium when challenged with PET, other natural polymers, BHET, and TPA. Because of the important role of *Vibrio* spp. in pathogenicity, regulatory networks have been well studied in this genus (48–50). These networks are known to be involved in surface attachment, biofilm formation, infection, toxin and secondary metabolite production, phage assembly, and others. Biofilm formation in *Vibrio* spp. is controlled by quorum sensing (QS), an integrated and highly complex network of multiple transcriptional regulators (e.g., VpsR, VpsT, HapR, H-NS, and AphA). The c-di-GMP signaling influences the planktonic-to-biofilm transition. In addition, cAMP-CRP signaling represses biofilm formation in *Vibrio* spp. (49, 50).

In this study, we showed that many of the above-mentioned regulatory circuits and their key regulators in DSM 21264 were affected by BHET and in part by TPA in planktonic cultures (Fig. 4 to 6). For instance, essential components of the QS signaling, like the low-cell density regulator, AphA, and the high-cell density regulator, HapR, were strongly attenuated by high concentrations of BHET. Like AphA and HapR, the autoinducer-I and 2 synthase genes (*luxM* and *luxS*), the cognate sensor genes (*luxN* and *luxQ*), and the transcription of the periplasmic binding protein (LuxP) were significantly affected in their transcription levels by 30 mM BHET (Fig. 5). QS thereupon regulates the c-di-GMP level in the cell. The major signal c-di-GMP is produced by diguanylate cyclases and degraded by specific phosphodiesterases that are directly regulated by HapR (50, 51). DSM 21264 has a very similar set of c-di-GMP cyclases and phosphodiesterases compared to *V. cholerae* or other well-studied *Vibrio* species. Many of the cyclase genes were differentially regulated at higher concentrations of BHET (Table S1; Fig. 4). The lack of sufficient cyclase transcription will most likely result in low intracellular levels of c-di-GMP that would not be sufficient to allow biofilm formation. This observation is in line with the failure to form biofilms in medium supplemented with >10 mM BHET (Fig. S6).

Within this framework, cAMP-CRP signaling is well known to be involved in the expression of inducible catabolic operons. Besides this function, cAMP-CRP signaling is also involved in processes related to infection and biofilm formation. Thereby, a specific adenylate cyclase catalyzes the formation of cAMP from ATP, whereas cAMP phosphodiesterases catalyze its breakdown (52, 53). The DSM 21264 cAMP-CRP signaling pathway is conserved as well, and its main regulatory protein is the CRP regulator. While CRP was highly transcribed under biofilm conditions, its transcription was in general much lower in the planktonic lifestyle. Notably, 5.0 and 30 mM BHET downregulated the CRP transcription even further, implying that BHET is interfering with this signaling cycle as well (Fig. 6). Overall, the interference with key regulatory networks implies that the degradation products of PET strongly induce stress responses in DSM 21264. This results in rapid changes in several metabolic pathways and is linked to different survival strategies.

Based on the above-made observations, we were able to establish a putative first model outlining the main genes and enzymes involved in PET degradation and metabolism and the regulatory circuits involved (Fig. 7). This preliminary model and our experimental data provide a foundation for future research on bacterial metabolism and nutritional signaling during life in the plastisphere. Since *Vibrio* spp. is found with high frequencies in the plastisphere, data from this study will help identify key factors involved in enriching for pathogens in this man-made habitat. Finally, this study will be useful for the design of bacterial strains for efficient plastic removal in the marine environment.

## MATERIALS AND METHODS

### Bacterial strains and growth conditions

Bacterial strains and plasmids used in this study are summarized in Table 1. *Vibrio* sp. was cultured either at 22°C or 28°C in artificial seawater medium (28.13 g/L NaCl, 0.77 g/L KCl, 1.6 g/L CaCl_2_ × 2H_2_O, 4.8 g/L MgCl_2_ × 6 H_2_O, 0.11 g/L NaHCO_3,_ 3.5 g/L MgSO_4_ × 7 H_2_O, 10 g/L yeast extract, and 10 g/L tryptone) or in AS medium 1:10 diluted (1 g/L tryptone and 1 g/L yeast extract) and either CMC (1% wt/vol), chitin (1% wt/vol), alginate (1% wt/vol), PET powder (1% wt/vol), TPA (1 mM), or BHET (0.5, 5 and 30 mM) was added. Additional growth experiments were implemented in M9 medium (Na_2_HPO_4_ 33.7 mM, KH_2_PO_2_ 22 mM, NaCl 51.3 mM, NH_4_Cl 9.35 mM, MgSO_4_ 1 mM, biotin 1 µg, thiamin 1 µg, EDTA 0.134 mM, FeCl_3_-6H_2_O 0.031 mM, ZnCl_2_ 0.0062 mM, CuCl_2_ 2H_2_O 0.76 µM, CoCl_2_ 2 H_2_O 0.42 µM, H_3_BO_3_ 1.62 µM, and MnCl_2_ 4H_2_O 0.081 µM).

*E. coli* was grown at 37°C aerobically in the LB medium (10 g/L tryptone, 5 g/L yeast, and 5 g/L NaCl) and supplemented with the appropriate antibiotics.

For the observation of the phenotypical reaction and halo formation of DSM 21264 to various concentrations of BHET, LB agar plates (10 g/L tryptone, 5 g/L yeast, 5 g/L NaCl, and 15 g/L agar) were prepared. After autoclaving, the respective amount of BHET was added to reach final concentrations of 0.5 mM, 5.0 mM, 10 mM, and 30 mM in the respective plate. Five microliters of DSM 21264 OD_600_ = 1 was added and incubated for 24 h at 28°C.

### Fluorescence imaging analysis of biofilms

To observe biofilm formation of DSM 21264 on plastic surfaces, cells were inoculated at OD_600_ = 0.05 in the ASW medium and incubated in 6-well cell culture plates (Nunc cell culture plate, catalog no. 130184; Thermo Fisher Scientific, Waltham, MA) at 22°C or 28°C at 80 rpm shaking. After incubation, the foil was washed in PBS buffer and placed into µ-slide eight-well (ibiTreat. catalog no. 80826, ibidi USA, Inc., Fitchburg, Wisconsin). Cells were stained using the LIVE/DEAD BacLight bacterial viability kit (Thermo Fisher Scientific, Waltham, MA, USA).

Cells were visualized using the confocal laser scanning microscope (CLSM) Axio Observer.Z1/7 LSM 800 with Airyscan (Carl Zeiss Microscopy GmbH, Jena, Germany) and a C-Apochromat 63 x/1.20W Korr UV VisIR objective. Settings for the microscope are presented in Table S5. For the analysis of the CLSM images, the ZEN software was used (version 2.3; Carl Zeiss Microscopy GmbH, Jena, Germany). For each sample, at least three different positions were observed, and one representative CLSM image was chosen.

### Preparation for scanning electron microscopy

Strains and polymers were incubated as described above, and after incubation, fixed overnight in 1% PFA in 50 mM cacodylate buffer, pH 7. The following day, samples were incubated in 0.25% GA in 50 mM cacodylate buffer, pH 7 overnight. For drainage samples, the samples were incubated in 30%, 50%, and 70% of ethanol in the following order, each for 20 min. An additional incubation in 70% ethanol was performed overnight. Drainage continued with sample incubation in 80%, 96%, and 100% of ethanol for 20 min and 100% of ethanol for 30 min. Critical point drying was performed using Leica EM CPD300, washed 18 x with CO_2_, followed by coating in a thin carbon layer using Sputter Coater LEICA EM ACE600. Microscopy was performed at the scanning electron microscope LEO 1525 using Software SmartSEM V06.00.

### Sample preparation for RNA-seq and analysis

Precultures of DSM 21264 were inoculated at OD_600_ = 0.05 in ASW medium (4 mL/well) in 6-well cell culture plates (Nunc cell culture plate, catalog no. 130184; Thermo Fisher Scientific, Waltham, MA). To each well, either PE or PET foil (35 mm diameter) was added and incubated for 8 hours at 22°C shaking at 80 rpm. The foil was washed in PBS buffer to get rid of planktonic cells, and then cells were scratched off the surface. Two milliliters of 20% stop mix consisting of 95% ethanol and 5% phenol was added to the cells, and the mixture was centrifuged for 20 minutes at 4°C. The supernatant was discarded, the pellet was washed three times in PBS buffer, and afterward immediately frozen in liquid nitrogen and stored at −70°C.

The liquid cultures were inoculated at OD_600_ = 0.05 in diluted artificial seawater. Respective C-source (CMC, PET powder (particle size 300 µm max; >50% crystallinity, product no. ES30-PD-000132), chitin and alginate (1% wt/vol) and BHET concentrations ranging from 0.5 mM, 5 mM, and 30 mM as well as 1 mM TPA) was added and incubated shaking at 28°C at 130 rpm for 24 hours. As negative controls, no additional carbon source and DMSO controls were prepared. Cells were harvested and cell pellets formed.

Harvested cells were re-suspended in 800 µL RLT buffer (RNeasy Mini Kit, Qiagen) with β-mercaptoethanol (10 µl m^L−1^), and cell lysis was performed using a laboratory ball mill. Subsequently, 400 µL RLT buffer (RNeasy Mini Kit Qiagen) with β-mercaptoethanol (10 µl m^L−1^) and 1,200 µL 96% (vol/vol) ethanol were added. For RNA isolation, the RNeasy Mini Kit (Qiagen) was used as recommended by the manufacturer, but instead of RW1 buffer, RWT buffer (Qiagen) was used in order to isolate RNAs smaller than 200 nucleotides also. To determine the RNA integrity number, the isolated RNA was run on an Agilent Bioanalyzer 2100 using an Agilent RNA 6000 Nano Kit, as recommended by the manufacturer (Agilent Technologies, Waldbronn, Germany). Remaining genomic DNA was removed by digesting with TURBO DNase (Invitrogen, Thermo Fischer Scientific, Paisley, UK). The Illumina Ribo-Zero plus rRNA Depletion Kit (Illumina Inc., San Diego, CA, USA) was used to reduce the amount of rRNA-derived sequences. For sequencing, the strand-specific cDNA libraries were constructed with an NEBNext Ultra II Directional RNA library preparation kit for Illumina and the NEBNext Multiplex Oligos for Illumina (96) (New England BioLabs, Frankfurt am Main, Germany). To assess the quality and size of the libraries, samples were run on an Agilent Bioanalyzer 2100 using an Agilent High-Sensitivity DNA Kit, as recommended by the manufacturer (Agilent Technologies). The concentration of the libraries was determined using the Qubit dsDNA HS Assay Kit, as recommended by the manufacturer (Life Technologies GmbH, Darmstadt, Germany). Sequencing was performed on the NovaSeq 6000 instrument (Illumina Inc., San Diego, CA, USA) using the NovaSeq 6000 SP Reagent Kit (100 cycles) and the NovaSeq XP 2-Lane Kit v1.5 for sequencing in the paired-end mode and running 2 × 61 cycles. For quality filtering and removing of remaining adapter sequences, Trimmomatic-0.39 (Bolger et al., 2014) and a cutoff phred-33 score of 15 were used. The mapping against the reference genome of *V. gazogenes* DSM 21264^T^ was performed with Salmon (v 1.10.2) (54). As mapping backbone, a file that contains all annotated transcripts excluding rRNA genes and the whole genome of the reference as decoy was prepared with a k-mer size of 11. Decoy-aware mapping was done in the selective-alignment mode with “–mimicBT2,” “–disableChainingHeuristic,” and “–recoverOrphans” flags as well as sequence and position bias correction and 10,000 bootstraps. For–fldMean and–fldSD, values of 325 and 25 were used, respectively. The quant.sf files produced by Salmon were subsequently loaded into R (v 4.3.2) (55) using the tximport package (v 1.28.0) (56). DeSeq2 (v 1.40.1) was used for normalization of the reads, and fold change shrinkages were also calculated with DeSeq2 (57) and the apeglm package (v 1.22.0) (58). Genes with a log2-fold change of +2/–2 and a p-adjust value <0.05 were considered differentially expressed.

Fastp (59) was used to remove sequences originating from sequencing adapters and sequences of low-quality sequences. Reads were then aligned to the *V. gazogenes* DSM 21264 reference assembly (60) using BWA mem. Differential expression analysis was carried out with DESeq2 (57). A gene was considered significantly differentially expressed in a comparison if the corresponding false discovery rate (FDR) was smaller or equal to 0.05 and the absolute log2-fold change (|log2FC|) was larger than 2. All software was used with standard parameters.

Sequence data reported in this publication have been submitted to the European Nucleotide Archive (ENA). They are publicly available under accession PRJEB80907.

Key genes are presented in Fig. 4 to 6. The size of the circles and color intensity were calculated based on the transcriptome hits per gene (absolute values) and scaled using log-10-fold changes to allow better scaling.

### Cloning and expression of *V. gazogenes* UlaG

*UlaG* (NCBI Ref. Seq. WP_072962133.1) was cloned into pET21a(+) vector (Novagen/Merck) using restriction sites *Nde*I and *Xho*I in front of C-terminal 6 x HisTag. Primers were designed using SnapGene (GSL Biotech LLC, San Diego CA, United States), and PCR was performed using parameters indicated by the design tool. After PCR cleanup, 7 µL DNA (≈30 ng/µL) was mixed with 1 µL of T4 Ligase Buffer (10 x), 1 µl of T4 Ligase (400 U/µL), and 1 µl of purified pET21a(+) plasmid and incubated overnight at 6°C. The ligation construct was transformed into competent *E. coli* DH5α cells, and positive clones were identified via DNA sequencing (Microsynth seqlab). Positive sequenced plasmids were isolated and transformed into competent *E. coli* T7 Shuffle cells.

### Protein production and purification

For protein production, cells were overexpressed by growing inoculated cultures with T7 SHuffle harboring pET21a(+)::*UlaG* or BL21(DE3) harboring pET28a(+)::*pet6* at 37°C to OD_600_ = 0.8. Cells were induced with IPTG to final concentrations of 0.4 mM and incubated at 22°C overnight until cells were harvested by centrifugation at 5,000 × *g*.

For protein purification, the cell pellet was resuspended in lysis buffer (50 mM NaH_2_PO_4_, 300 mM NaCl, 10 mM imidazole, pH 8.0) and sonicated for cell disruption with Ultrasonic Processor UP200S by *Hielscher* with amplitude 70% and cycle duration of 0.5 units. Afterward, the proteins harboring a sixfold C-terminal histidine tag were purified with nickel-ion affinity chromatography using Ni-NTA agarose (Qiagen, Hilden, Germany). The elution buffer was exchanged against 0.1 mM potassium phosphate buffer pH 8.0 in a 30 kDa Amicon Tube (GE Health Care, Solingen, Germany).

### Measurement of PET and BHET degradation

Precultures of DSM 21264 were inoculated at OD_600_ = 0.05 in 20 mL M9 medium supplemented with 5 mM of glucose and additional 5 mM of BHET. Flasks were incubated by shaking at 28°C and 130 rpm for 6 days. Each day, 1 mL of each sample was taken and measured at the UHPLC. As a negative control, the medium was incubated under the same conditions with *E. coli* Dh5α, and samples were taken at the same time points.

For the detection of PET powder and foil degradation, DSM 21264 was incubated as described above in six-well plates, and also 0.5 mL of sample was taken each day and prepared for UHPLC measurements.

For enzymatic BHET degradation, purified enzyme in varying concentrations was incubated with 300 µM of BHET for 24 h and samples were prepared for UHPLC.

Respective supernatants were prepared and measured at the UHPLC following protocols described previously (61, 62).

### Cloning promoter fusion of *adhE*

The promoter of *adhE* (AAC977_10260) was identified using the software tool published at softberry.com (BPROM - Prediction of bacterial promoters) and cloned into the pBBR1-MCS-1 vector (39) carrying amCyan in the multiple cloning site using restriction enzymes *Sac*I and *Xba*I.

Primer design, PCR, ligation, and transformation into competent *E. coli* DH5α cells were performed as described above. Positive constructs were transformed into competent *E. coli* WM3064 cells. Since electroporation did not result in sufficient colonies, the plasmid was conjugated via biparental conjugation into DSM 21264. Cells were grown in overnight cultures. One milliliter of donor strain (*E. coli* WM3064 carrying the respective plasmid) and of receptor strain (DSM 21264) were mixed at ratio 1:1 and centrifuged at 5,000 rpm for 8 min. Cells were washed three times in LB medium, resuspended after the final centrifugation step in 150 µL of the LB medium, and a spot was given onto an LB agar plate supplemented with 300 µM DAP. The plate was incubated overnight at 28°C, and the spot was washed off. Dilutions ranging from 1:1 to 1:5 were prepared and plated onto ASWM agar plates supplemented with 25 µg chloramphenicol and 25 µg kanamycin. Colonies were detectable after 2 to 3 days of incubation and inoculated in the liquid medium.

### Promoter fusion measurements

For the identification of the influence of ethylene glycol on the expression of *adhE,* DSM 21264 carrying promoter fusion construct pBBR1MCS-1_*adhE_amcyan* as well as wild-type strain were inoculated at OD_600_ = 0.05 in M9 medium with 5 mM glucose. Cells were incubated in 48 well-plates (1 mL/well) (Nunc cell culture plate, catalog no. 150687; Thermo Fisher Scientific, Waltham, MA, USA) for 48 h at 130 rpm shaking. For the detection of the influence of ethylene glycol, 5 mM was added, and strains were grown in the presence with and without EG. After incubation, optical density and fluorescence of cyan (450;496) were measured with the Synergy HT plate reader using Gen5 software (Biotek, Winooski, USA). The average was evaluated, and fluorescence was divided by optical density to detect fluorescence units.

### Plasma activation of PE and PET foil

For plasma activation, an atmospheric air plasma system from Plasmatreat GmbH (Steinhagen, Germany) was used. The atmospheric pressure plasma was produced by a generator FG5001 with an applied working frequency of 21 kHz, generating a non-equilibrium discharge in a rotating jet nozzle RD1004 in combination with the stainless-steel tip No. 22826 for an expanded treatment width of approximately 22 mm. Additionally, the jet nozzle was connected to a Janome desktop robot type 2300N for repetitious accuracy regarding treatment conditions. The process gas was dry and oil-free air at an input pressure of 5 bar in all experiments.

## Data Availability

Sequence data reported in this publication have been submitted to NCBI/ENA. The raw reads of the 42 sequencing runs are publicly available under accession PRJEB80907 at the European Nucleotide Archive (ENA). The newly established genome sequence of *Vibrio gazogenes* DSM 21264 is available under accession numbers CP151640 and CP151641 at the NCBI/GenBank.

## SUPPLEMENTAL MATERIAL

Supplemental tables and figures spectrum.00181-25-s0001.docx Tables S2 to S5 and Fig. S1 to S8.

Table S1 spectrum.00181-25-s0002.xlsx Results of all 42 RNA seq experiments.

ASM does not own the copyrights to Supplemental Material that may be linked to, or accessed through, an article. The authors have granted ASM a non-exclusive, world-wide license to publish the Supplemental Material files. Please contact the corresponding author directly for reuse.
